# Carbon Nanomaterials in Seed Priming: Current Possibilities

**DOI:** 10.1021/acsomega.4c07230

**Published:** 2024-10-29

**Authors:** José Daniel da Silva Fonseca, Ewa Wojciechowska, Joanna Kulesza, Bráulio Silva Barros

**Affiliations:** † Programa de Pós-graduação em Ciência de Materiais, Centro de Ciências Exatas e da Natureza-CCEN, Universidade Federal de Pernambuco, Av. Prof. Morais Rego, 1235-Cidade Universitária, Recife, Pernambuco 50670-901, Brasil; ‡ Gdansk University of Technology, Faculty of Civil and Environmental Engineering, Narutowicza 11/12, 80-233 Gdansk, Poland; § Departamento de Química Fundamental, Centro de Ciências Exatas e da Natureza-CCEN, Universidade Federal de Pernambuco, Av. Prof. Morais Rego, 1235-Cidade Universitária, Recife, Pernambuco 50670-901, Brasil; ∥ Departamento de Engenharia Mecânica, Centro de Tecnologia e Geociências-CTG, Universidade Federal de Pernambuco, Av. Prof. Morais Rego, 1235-Cidade Universitária, Recife, Pernambuco 50670-901, Brasil

## Abstract

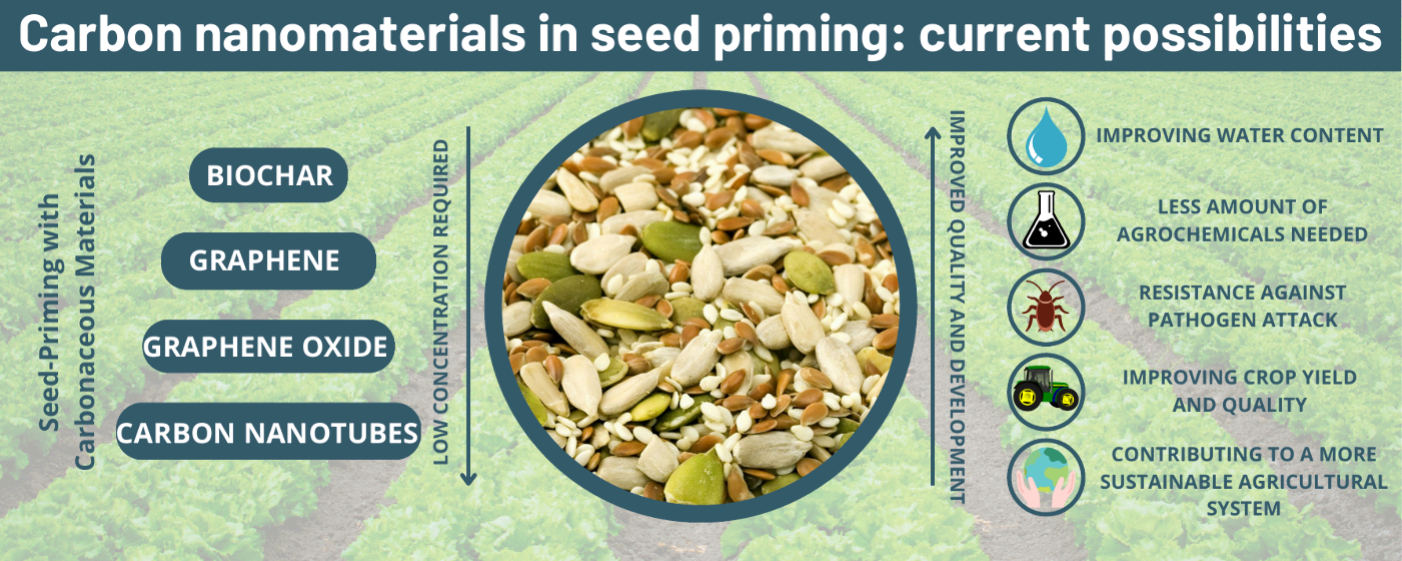

The prevailing agricultural system has become deeply ingrained and insufficient due to outdated practices inherited from the Green Revolution, necessitating innovative approaches for sustainable agricultural development. Nanomaterials possess the potential to significantly improve the efficient utilization of resources while simultaneously encouraging sustainability. Among these, carbonaceous nanomaterials have found diverse applications in agriculture, exhibiting remarkable capabilities in this domain. Notably, using biowaste to produce these materials makes them both cost-effective and environmentally friendly for seed priming. Seed priming is a technique that can potentially enhance germination rates and stress tolerance by effectively regulating gene pathways and metabolism. This review provides a comprehensive summary of recent progress in the field, highlighting the challenges and opportunities of applying carbonaceous materials in seed priming to advance sustainable agriculture practices. The existing reviews provide a general overview of using carbonaceous materials (graphene and derivatives) in agriculture. Yet, they often lack a comprehensive examination of their specific application in seed-related contexts. In this review, we aim to offer a detailed analysis of the application of carbonaceous materials in seed priming and elucidate their influence on germination. Furthermore, the review shows that crop response to carbonaceous nanomaterials is linked to material concentration and crop species.

## 1 Introduction

The agricultural sector is the central pillar of global food production and plays a vital role in sustaining human life. However, the current agricultural system faces numerous challenges that decrease crop yield production.^1^ These challenges are related to the effects of global climate change, deteriorating soils, attacks of pests and diseases, and low efficiency in the use of agrochemicals that significantly interfere with agricultural production.^2^

Future demands for food are predicted to be 50–80% higher to supply the worldwide population by 2050.^3^ The current agricultural system has achieved its maximum potential, and the ongoing practices are not environmentally sustainable. These practices are inherently inefficient and lead to inadmissible environmental depreciation. New agricultural approaches are needed to overcome these challenges and pave the way to a more efficient and sustainable agricultural system.^4^

**Figure 1:**
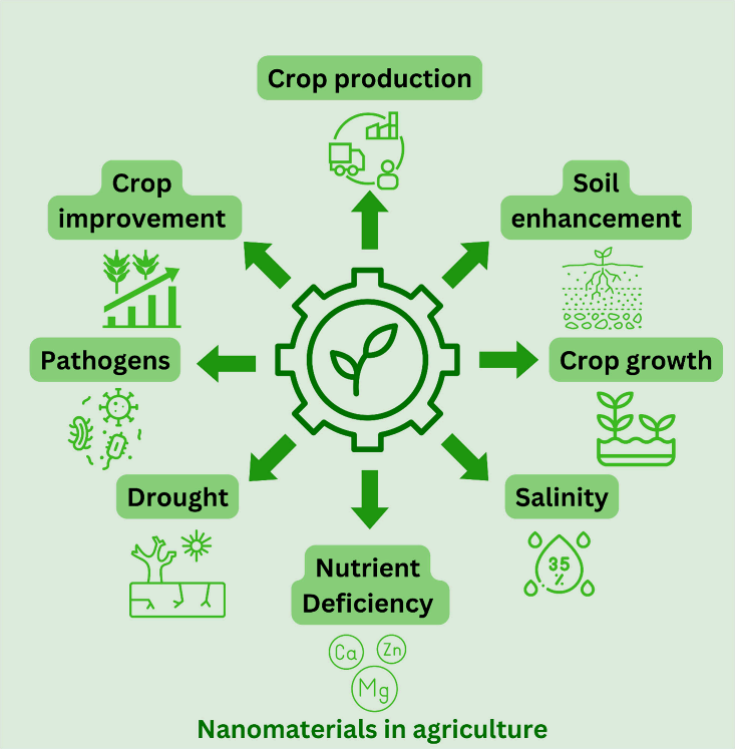
Potential application ways of nanomaterials in agriculture.

Nanotechnology has emerged as a potential new approach to an agri-tech revolution which may surpass the present problems.^2^ The applications of nanomaterials include nano pesticides, nano fertilizers, slow-release systems, and nanomaterials for soil and water remediation. Utilizing these pathways for nanomaterials, the applied amounts of agrochemicals can be reduced while increasing crop production and safety.^5^Figure 1 shows some agriculture areas the nanomaterials can be applied to improve production and minimize losses.

Nanomaterials, notably carbon-based ones, have a range of applications in agricultural crops. The main application is crop protection and biostimulation against biotic and abiotic effects. These fields of application are important to the agricultural industry and have gained particular attention due to the future challenges of increasing food production. The use of nanomaterials can contribute to crop nutrition and protection, disease management, and environmentally friendly production methods.^6−8^

Recent studies have revealed that carbonaceous materials affect seeds, seedlings, and plant growth and development.^9^ The influence of carbonaceous materials (graphene, graphene oxide, and carbon nanotubes) has a dose- and species-dependent manner, which can affect positively or negatively the growth evolution.^10−15^ In addition, they can act as biostimulators under stressful conditions, improving germination rates, seedling vigor, gene pathways, and plant growth. These effects are strongly dose-dependent and can vary from one crop variety to another.^13^ The size of nanomaterials is crucial because it determines their ability to penetrate the plant seed coat and affects germination and plant growth. This penetration can allow water uptake inside the seeds, improving germination.^16^

Seed-priming with carbon-based nanomaterials is a technique that provides a range of benefits for agricultural systems.^17^

Seed priming with carbonaceous materials can be employed to enhance seed protection during storage, promote germination and germination uniformity, and support plant growth.^9,10^ Additionally, nanopriming technique can bolster crop resistance to abiotic and biotic stressors, potentially reducing the need for pesticides and fertilizers.^6^ The use of carbonaceous nanomaterials for seed priming offers significant advantages compared to the traditional priming techniques, due to the small size of the nanoparticles, which enables them to rapidly penetrate seeds by crossing biological barriers.^18^ Their catalytic properties, combined with unique physicochemical characteristics, allow these nanoparticles to influence various metabolic processes and modulate metabolic pathways during the early stages of germination.^19,20^

Upon crossing the cell wall, nanoparticles can generate reactive oxygen species, which in turn trigger the activation of signaling molecules and proteins involved in stress resistance mechanisms.^19^ Additionally, carbonaceous nanomaterials can be functionalized with metallic nanoparticles, such as those with antimicrobial properties, to provide enhanced protection to seeds against pathogens. Another application of carbonaceous materials is to improve water retention in seeds.^21^ By modifying the chemical groups on the surface of these nanomaterials, their interaction with water molecules can be enhanced, thereby increasing water retention during germination. This property is particularly beneficial in mitigating drought stress, as it helps seeds maintain adequate hydration under conditions of limited water availability.

In 2021, Carvalho and colleagues conducted a study analyzing 996 scientific papers about nanomaterials used in agriculture.^22^ Their research yielded intriguing findings regarding the application of nanomaterials to seeds. Surprisingly, they found that only 10% of these studies focused on carbon-based materials like carbon nanotubes, graphene, and fullerenes, which were applied in general in agriculture. More than 65% of the analyzed articles were related to the exposure of the roots to the nanomaterial. Additionally, just 13% of the studies explored the use of nanomaterials (metal, metal oxides, and carbon-based) directly on seeds. This highlights the need to investigate further this method (seed-priming) and materials (carbon-based) that have received limited attention. Priming seeds with nanomaterials can improve protection against fungal, bacterial, nematoid, and virus attacks; it can also directly affect germination and increase resistance to abiotic conditions, which can help overcome the excessive use of agrochemicals.^17,23^ The application of carbon-based nanomaterials for seed priming is an emerging field of study, and early research has already demonstrated encouraging outcomes.^24−27^

The field of carbonaceous materials as seed priming agents is at an early stage of development. The majority of the literature reports are limited to the general presentation of nanomaterials. The discussion of the dose-dependent response of carbonaceous materials like graphene and its relatives has been rarely mentioned, and the use of graphene-like materials has recently emerged. Details about the influence of carbonaceous materials on germination, the improvement of resistance against biotic and abiotic stress, and biostimulation, for example, are still rare. However, as the need for more environmentally friendly agriculture grows, advanced studies have been carried out to improve current agricultural technologies.

This review article focuses on the prominent class of carbonaceous materials, including those resembling graphene, which finds application in seed priming. Therefore, this review will not provide an exhaustive analysis of the extensive body of literature on the use of nanomaterials in agriculture. Instead, it will specifically focus on carbon-based materials and their potential to alleviate stress conditions associated with biotic and abiotic factors, particularly through their application to seeds.

## 2 Innovative Nanomaterial Strategies in Agriculture to Combat Abiotic and Biotic Stress and Boost Crop Yields

In recent years, nanotechnology has emerged as a turning point in overcoming the challenges faced by the current agricultural system. Nanotechnology is related to the synthesis and manipulation of materials in a nanoscale manner; frequently, nanomaterials are characterized to have one of its dimensions in the range of 1–100 nm. Nanomaterials, with their unique properties and high surface area, have many potential applications in agriculture, such as nanofertilizers, nanopesticides, nutrition, crop protection, biostimulants under stressful conditions, and environmental remediation. Some of these applications are highlighted in this section.

To face the climate challenges and meet the future global demands for food and energy, engineered nanomaterials can offer a way out of the current problem of low efficiency.^28^ Unquestionably, there has been a notable increase in the use of nanotechnology in plant protection and crop yield, providing a hopeful possibility for increased agricultural productivity. A major goal of sustainable agriculture is to ensure high production and support crop adaptation to the effects of climate change, including extreme temperatures, water scarcity, salinity, pathogens, diseases, and environmental contamination by dangerous metals, while protecting the environment.^29^ Given these facts, it is necessary the development of agri-tech tools that improve crop protection against biotic and abiotic factors which will lead to an increase in crop production yield. Agri-tech tools based on nanotechnology can improve the quality and yield of crop production in a sustainable way, ensured by a minimal use of resources.^1^

A critical aspect often overlooked in managing agricultural systems is crop production, which is the foundation for global food security. However, crop production yield is influenced by a broad spectrum of factors, encompassing both biotic and abiotic elements.^1^ These factors create stressful conditions that can lead to a decrease in crop production and quality. Approximately 50% of the average global agricultural productivity is estimated to be reduced due to the impact of biotic and abiotic stressors.^30^ Biotic factors are related to living organisms, such as pests, diseases, and weeds. In contrast, abiotic factors are related to nonliving organisms, like drought, heat, salinity, cold, and flooding.^31^

**Figure 2:**
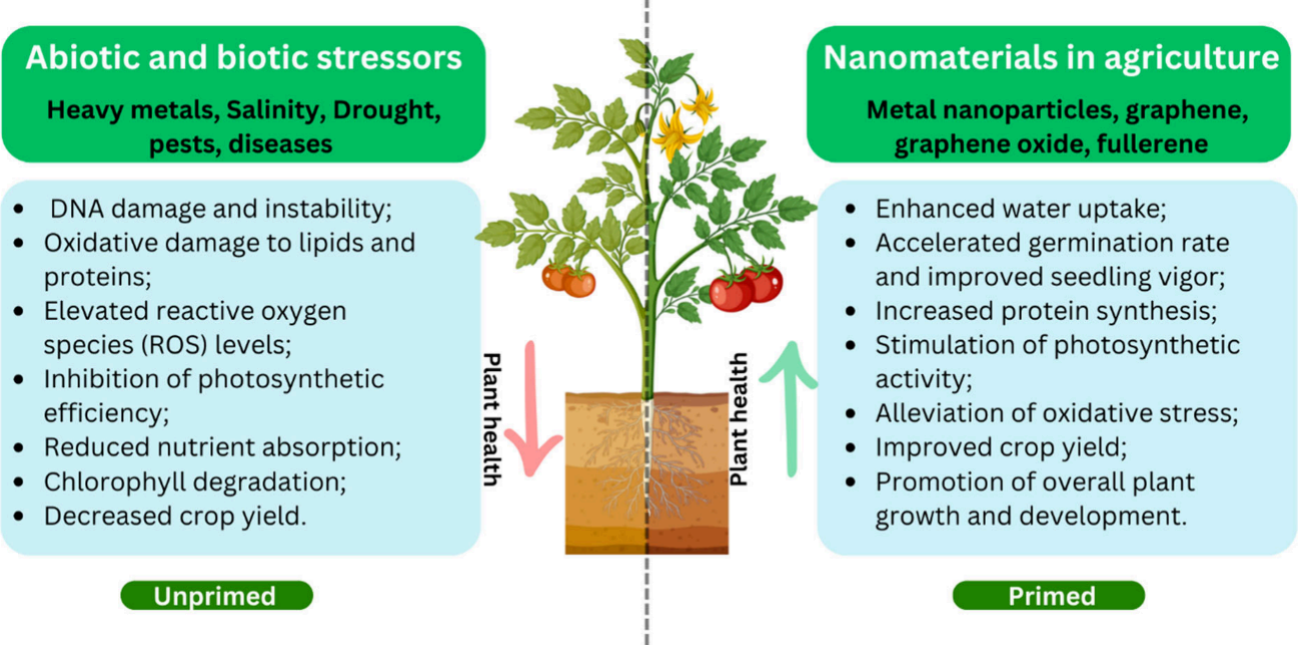
Effects of abiotic and biotic stresses on plants and the use of nanomaterials for alleviation.

To ensure maximum agricultural yield, integrated pest management, disease-resistant crop types, and efficient weed control techniques must be used to manage and mitigate the negative impacts of biotic and abiotic variables. The response of a plant to stressful conditions involves its metabolic pathways, which include signaling agents that work to mitigate the adverse effects.^32^ Suzuki and co-workers reported the effects of the combinations of more than one effect (abiotic or biotic) on the development of crops and how it can impact production, reiterating the importance of the study of these combined stressors and their mitigation given an increased production and quality of crops.^31^ Nanotechnology has emerged as a promising device for advancing sustainable and productive agriculture. Nanomaterials offer various avenues to address the challenges of both abiotic and biotic conditions. Figure 2 illustrates the key effects of biotic and abiotic stresses on plants, as well as the alleviating impacts of nanomaterial applications. For instance, graphene has been utilized as a biostimulant to alleviate the detrimental effects of salinity on melon production, showcasing its potential to enhance crop resilience through a salinity condition.^13^ The infection caused by the pathogen *Fusarium graminearum* was successfully mitigated through the utilization of silver nanoparticles supported on graphene oxide.^33^ Carbon nanotubes have shown potential in alleviating drought stress by acting as water channels and supporting seed coat integrity, while graphene oxide has demonstrated its ability to retain water through the interaction between oxygen groups on its surface and water molecules in the soil.^16,34^

Crop production is greatly affected by pest attacks, which cause crop losses. Many types of pesticides have been used to combat the effects of pathogens. To face the stress caused by pathogen attacks, a response in the protective crop system can be produced by biostimulation, such as activation of the plant enzymatic system, metal uptake, water deficit, hormonal regulation, and stress gene expression.^28^

Nanoparticles and nanopesticides can regulate and induce these pathways, increasing protection against biotic factors.^35^ Recent studies in applying nanomaterials in agriculture have shown that using nanopesticides can improve crop protection with a minimum amount of resources.^7^ The capability of nanomaterials to enhance crop yield and protect against pathogens has been demonstrated in the literature.^36^ Acharya and co-workers reported the green synthesis of nanoparticles using onion extracts as reducing agents. In the study, silver and gold nanoparticles were successfully synthesized and characterized. The nanoparticles were used as a priming agent for aged onion seeds. The results showed that the nanoparticles were internalized into the seeds which might have helped improve water uptake, germination, and growth, leading to an increase in crop yield.^37^ Silver nanoparticles (AgNPs) were applied to rice seeds (*Oryza sativa* L.) under saline conditions.^18^ The application of AgNPs stimulated the generation of reactive oxygen species (ROS), which in turn preconditioned the seeds to better withstand stress by activating metabolic pathways that mitigate stress effects. The study demonstrated that AgNP treatment enhanced germination, seedling vigor, biomass accumulation, and root length. Furthermore, the treated seeds exhibited moderate resistance to the pathogen Magnaporthe oryzae, which causes significant damage to rice crops and severely reduces yield. Another study investigating the application of AgNPs to maize seeds (*Zea mays* L.) demonstrated that primed seeds exhibited enhanced seedling growth under saline, cold, and drought conditions, as well as when exposed to these stress factors simultaneously.^38^ Nanoparticles can be used to protect against abiotic effects like salinity. In the work of Latef and co-workers, titanium dioxide nanoparticles were used to ameliorate soil salinity.^39^ Malandrakis and co-workers reported the fungitoxicity of different NPs in seven plant pathogenic fungal strains. They demonstrated the potential of NPs in combating plant pathogenic fungi, in which Cu-NPs and Zn-NPs were the most effective.^40^ There is a wide range of information in the literature about the use of nanomaterials in crop protection.^28,35,36,41,42^

The Green Revolution has introduced tools that provided gains with minimal expansion of utilized areas. Synthetic fertilizers, particularly nitrogen-based compounds, provide essential nutrients to crops, boosting their growth and yields. This practice helped maximize the production potential of existing farmland. One of the most successful ways was the use of the nitrogen fertilizer provided by the Haber-Bosh process.^1^ Nitrogen fertilizers used in large amounts have serious environmental impacts since 50–70% of the nitrogen applied is lost by volatilization and leaching.^43^ Additionally, a wide variety of chemicals were introduced due to the varieties of crop responsiveness. This low efficiency leads to the implementation of environmentally friendly practices and improved efficiency of nutrient utilization. Nanofertilizers can increase production yield while meeting the global agenda for sustainable agriculture.

Quality and yield can be improved by nanofertilizers with increased effectiveness of nutrient utilization while production costs are reduced.^44^ Kabiri and co-workers reported a new carrier platform based on graphene oxide sheets.^45^ This material was used to load two micronutrients, zinc and copper. Experiments showed Zn and Cu uptake by wheat was higher compared to commercial fertilizers. Additionally, a composite based on biochar and graphene oxide has shown that micronutrient availability increased with less than 0.5% loss due to solubilization.^46^ Zhao et al. reported the potential application of carbon nanoparticles as a fertilizer for nutrient uptake, soil fertility, and crop growth of corn.^47^ Their principal findings were that the use of carbon nanoparticles enhanced the growth of corn in sand soil by improving the uptake of N, P, and K. Nitrogen, phosphorus, and potassium are macronutrients (NPK) important for many crops. Taking this into account, Rashid and co-workers suggest that Carbon-based slow-release fertilizers contribute to optimizing the uptake of NPK by plants by enhancing nutrient retention, minimizing losses, increasing availability, and ensuring a continuous nutrient supply during the entire crop growth period.^48^ The potential application of nanomaterials as nanofertilizers is evident from the reports, which can sustainably improve crop production while minimizing production costs. Utilizing nanomaterials as nano fertilizers is a pathway that can guide to an economical and sustainable solution for the current agricultural system regarding crop nutrition. These approaches can effectively overcome the barriers imposed by abiotic, biotic stress, and fertilizer use efficiency, enhancing plant resistance to these stressors and boostering the resilience and strength of the agricultural system in the face of climate change.

## 3 Seed Priming

**Figure 3:**
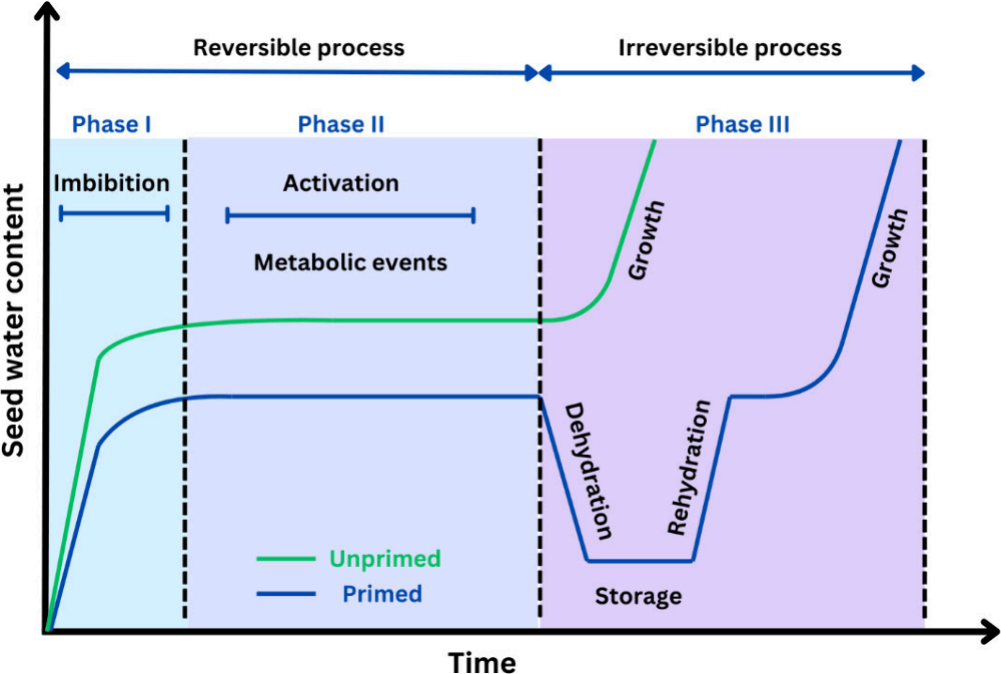
Representation of water uptake dynamics and critical phases of seed germination in primed and unprimed seeds.

Seed priming is a technique used to enhance the quality and performance of seeds, making them more resilient against various environmental stresses and diseases.^19^ This approach involves the submersion of seeds in water (hydropriming) or solutions with nutrients, hormones, and other substances that may confer any benefit to seed development.^6^ The mechanism involved in the seed priming process evokes several key steps. The exposure of a dry seed to a hydrated environment triggers various metabolic processes within the seed (Figure 3).

**Figure 4:**
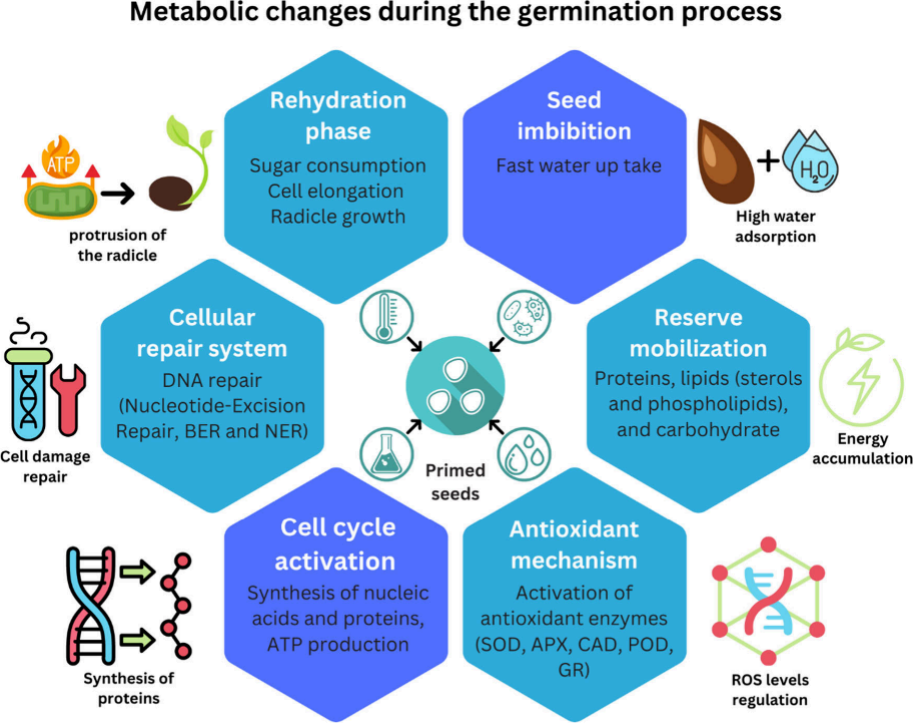
Metabolic modification during seed germination.

Germination in nondormant seeds typically occurs in three distinct phases: (I) imbibition, (II) the lag or activation phase, and (III) radicle protrusion or rehydration.^49,50^ During the first phase (imbibition), water enters the seed via adsorption.^51^ In the second phase (activation), metabolic processes initiate, involving a myriad of cellular events critical to early germination. These events include protein synthesis, enzyme activation, accumulation of sterols and phospholipids, antioxidant system activation, new mitochondria formation, and DNA repair.^19,20,52^ In seeds that have undergone priming, germination is halted during the second phase before radicle emergence. At this point, the seeds are dehydrated for storage, and drying at this stage does not harm seed viability.^53^ According to Di Girolamo,^54^ drying after the onset of radicle emergence causes significant damage to seed viability and vigor. The third phase, often referred to as rehydration, is characterized by rapid water uptake. During this phase, priming initiates cell division, and radicle protrusion marks the transition to active growth. At this stage, nucleic acid and ATP synthesis increase cellular energy, which drives sugar consumption by the embryo to fuel further development.^21,55^Figure 4 illustrates the metabolic and molecular changes that occur during seed germination.

### 3.1 Nanomaterials in Seed Priming

The use of nanomaterials in seed priming (seed nanopriming) is an emerging area of research that aims to enhance the efficiency of seed germination and seedling development. Nanomaterials applied in seed priming directly impact germination, yield, and stress tolerance. These facts are due to the ability of nanomaterials to modulate gene expression, plant metabolism, and nutrient uptake, which can directly influence germination and development aspects of plants.

Several types of nanomaterials have been used in seed priming. For example, Acharya et al. demonstrated that onion seeds treated with gold nanoparticles at a low concentration (5.4 ppm) enhanced germination at all development stages without any toxicity effect.^37^ Another similar study involving nanoparticles was performed by Maswada et al.^56^ In their work, iron oxide nanoparticles were used as a nanopriming agent in sorghum seeds under salinity stress,^56^ and it was observed that for seeds primed with 100 mg L^–1^ of *n*-Fe_2_O_3_, the salinity stress was alleviated and the seedling vigor index was improved.

### 3.2 Carbon Nanomaterials in Seed Priming

**Figure 5:**
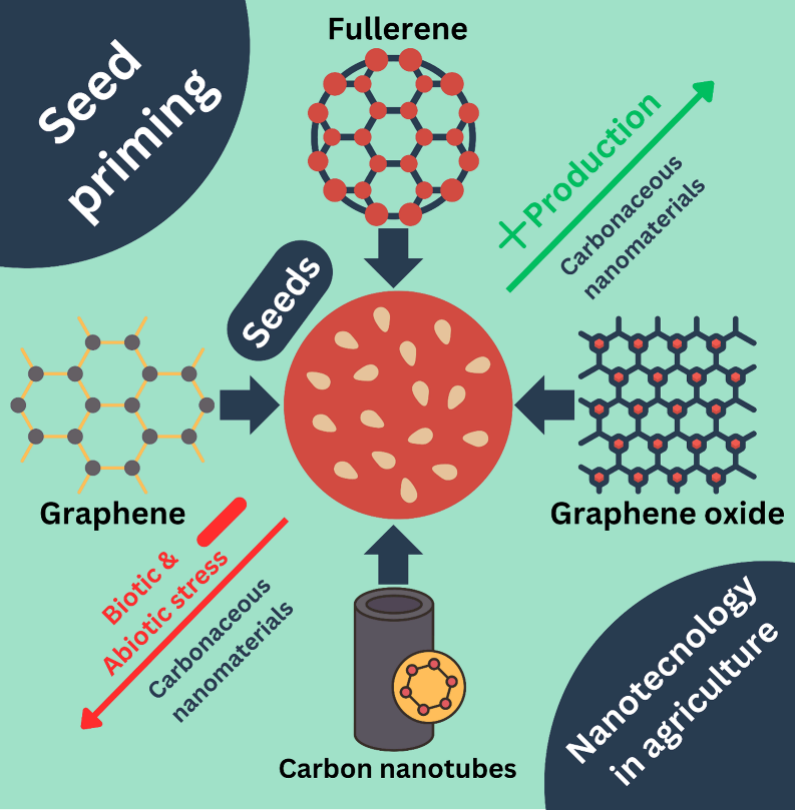
Carbonaceous nanomaterials for seed priming application.

The application of nanoparticles in seed priming is not limited to the use of metal nanoparticles. Khodakovskaya et al. performed one of the first works using nanomaterials to affect seed germination.^16^Figure 5 illustrates some carbonaceous materials that can be applied on seed priming. Their study demonstrated that carbon nanotubes could penetrate the rice seed and increase the water uptake content, improving the germination rate. In a different study performed by Zhang et al., biochar nanoparticles (BNPs) were used to promote the germination and seedling growth of tomatoes, rice, and reeds.^57^ According to their study, using BNPs promoted inhibition of shoot growth and biomass in reed; on the other hand, the improvement of shoot and root growth in rice was observed. Additionally, their work highlighted the toxicity that BNPs can have on plants in high concentrations.^57^

**Figure 6:**
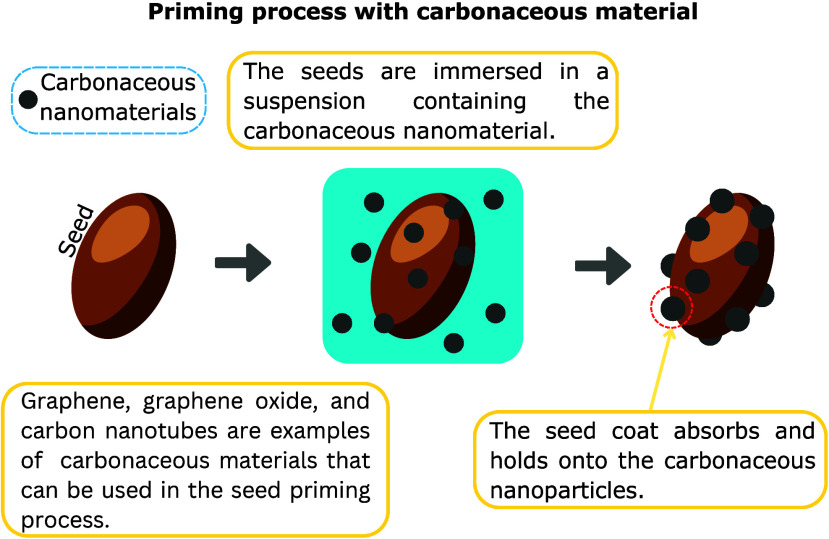
Representation of the process by which carbonaceous materials are applied as a coating on the seed surface.

In recent years, there has been an increased interest in researching innovative seed priming techniques that can improve the effectiveness of the process. Among promising nanomaterials, carbonaceous materials have emerged as promising alternatives in agriculture due to their unique physicochemical characteristics and capacity to interact with plant tissues.^58^Figure 6 illustrates the potential mechanisms by which carbon-based materials can prime seeds. The conventional approach for synthesizing carbonaceous materials such as graphene, graphene oxide, and reduced graphene oxide typically involves the use of oxidative and reductive agents that are detrimental to the environment. To address this issue, researchers have explored alternative methods for producing carbonaceous and graphene-like materials.^59^ One promising approach is the utilization of biowaste sources, including animal manure, leaves, and fruit peels, for the synthesis of these materials. This method can produce carbonaceous materials at a significantly lower cost through processes that are less dependent on hazardous chemicals.^60,61^ Several preparation techniques have been developed for this purpose, such as hydrothermal carbonization, pyrolysis, and chemical activation, which facilitate the transformation of biowaste into high-quality carbonaceous materials. These biowaste-derived materials offer a scalable, environmentally friendly, and rapid synthesis route, providing multifunctional materials while adding value to otherwise discarded organic waste.^59,62,63^ This green approach not only mitigates the environmental impact of traditional methods but also promotes the sustainable use of biowaste in advanced material production. Carbonaceous materials encompass many substances, from traditional forms like activated carbon and charcoal to advanced nanomaterials like carbon nanotubes and graphene-based materials.^9^ Their typically high surface area allows for better interactions with plant cells and tissues by facilitating the adsorption and release of nutrients, growth regulators, and other bioactive compounds.^27^ Carbonaceous nanomaterials are generally considered biocompatible and safe for plant use when applied in appropriate concentrations. However, assessing the specific nanomaterial potential impacts on the environment and human health is important. Without a doubt, incorporating carbonaceous elements into seed priming is a potential strategy for improving seed quality and seedling establishment. The following subsection reports on the application of carbon nanotubes, graphene, and graphene oxide as seed priming agents and their impact on the growth and production yield of many crops.

#### 3.2.1 Carbon Nanotubes

Carbon nanotubes (CNTs) are a class of materials that have generated widespread interest due to their remarkable properties and potential applications in various fields, including electronics, energy, medicine, and materials science. CNTs, which resemble a rolled-up sheet of graphene, a two-dimensional form of carbon, are cylindrical objects made of carbon atoms arranged in a certain pattern. CNTs come in two varieties: single-walled, made up of a single cylindrical tube, and multiwalled, which are made up of nested concentric tubes. A CNT can have a length of up to several micrometers or even millimeters with a diameter that commonly falls between a few and tens of nanometers.^64^

The extraordinary mechanical strength of CNTs, which is many times greater than that of steel while being significantly lighter in weight, is one of their most fascinating characteristics. Additionally, CNTs have exceptional electrical and thermal conductivity, making them desirable options for energy storage and electronics use. Additionally, CNTs have special optical and chemical capabilities that could be used in fields including medication delivery, catalysis, sensing, and agrotech applications.^64^

Pandey and co-workers^11^ tested the impact of multiwalled carbon nanotubes on the germination and mass production of two bioenergy crops, sorghum (*Sorghum bicolor* L.) and switchgrass (*Panicum virgatum* L.), under saline conditions. Bioenergy crops are well-known to be used in power generation like electricity, heat, and liquid fuels.^65^ In this study, the seeds of switchgrass and sorghum were sterilized by washing them with 70% ethanol for 2 min, and then they were washed twice with distilled water. The concentrations of multiwalled CNTs chosen in their study were 50 and 200 μg mL^–1^. The seeds were germinated in a Murashige and Skoog (MS) medium in the presence of CNTs. The results showed that applying CNTs to seeds significantly enhanced the seed germination rate of switchgrass and sorghum. The exposure of switchgrass and sorghum seeds to 50 μg mL^–1^ increased the germination rate by 20 and 22%, respectively, compared to untreated seeds. The effect of CNTs on the salt stress response of exposed sorghum and switchgrass was also studied. CNTs were able to lessen the toxicity of NaCl in a salted growth media, which was inhibiting seed germination.

The influence of CNTs on the germination and seedling growth of tomato (*Lycopersicum esculentum* Mill), onion (*Allium cepa* L.), turnip (*Brassica rapa* L.), and radish (*Raphanus sativus* L.) was studied by Haghighi and co-workers.^66^ The studies were conducted in a laboratory and greenhouse. Seeds were surface sterilized by submerging in a 50% (v/v) solution of sodium hypochlorite for 10 min, then rinsed once with sterile distilled water. Seeds were placed on top of a single sheet of filter paper inside Petri dishes and soaked with 2 mL of each CNT concentration. The CNT concentrations chosen for the experiments were 0, 10, 20, and 40 mg L^–1^. The results indicated that applying CNTs at 10–40 mg L^–1^ improved the germination percentage of tomatoes and onions, but not of radish or turnips. The observed decrease in radish and turnip germination suggests that applying a high concentration of CNTs may have a toxic effect on germination.

In another study, reported by Srivastava et al.,^67^ the authors investigated the influence of functionalized multiwalled carbon nanotubes (MWCNT) on wheat (*Triticum aestivum*), maize (*Zea mays*), peanut (*Arachis hypogaea*), and garlic bulb (*Allium sativum*). MWCNTs were functionalized with carboxylic acid groups. The seeds of wheat, maize, peanuts, and garlic were only washed with deionized water. The MWCNT concentrations chosen were 20 and 50 μg mL^–1^. The seeds were soaked in the MWCTN solution overnight in the absence of light. In the study, a significant positive influence on root and shoot elongation was observed for all seeds compared to the control. The results indicated that all the studied seeds (wheat, maize, peanut, and garlic seedlings) were affected by water-soluble, multiwalled carbon nanotubes. Sharma et al.^68^ studied the effect of single-walled carbon nanotubes (CNTs) on pearl millet seeds. The effect was evaluated based on the seed germination rate, photosynthetic pigmentation, proline concentration, and indole acetic acid content of pearl millet (*Pennisetum glaucum*). The CNT concentrations chosen for the study were 30, 60, 90, 120, and 150 ppm. In the experiments, carbon nanotubes functionalized with carboxylic acid groups were used. The pearl millet seeds were cleaned with 20% [v/v] Extran for 3 to 4 min. Then, the seeds were washed with distilled water three times. The surface of the seeds was disinfected with a laminar air flow hood fitted with UV light using a 0.1% HgCl_2_ solution for at least 3 min, and then the seeds were washed three times with autoclaved distilled water. The seeds were soaked in the prepared carbon nanotube suspension for 24 h at 100 rpm in a shaking incubator. For seeds treated with 90 ppm of MWCNTs, higher germination was observed (80%). MWCNTs of concentration higher than 90 ppm (120 and 150 ppm) cause a decrease in seed germination. This fact suggests that higher doses of MWCNTs may cause less water permeability because graphene sheets can block the coat pores of the seed.^68^ Krumova and co-workers^69^ conducted research on the use of Pluronic P85 polymer-functionalized carbon nanotubes as a seed priming agent for pea (*Pisum sativum*) seeds. Single-walled carbon nanotubes (SWCNTs) were utilized in the following concentrations: 50, 100, and 300 mg L^–1^. The seeds of *Pisum sativum* were soaked in the P85-SWCNTs under continuous shaking at 25 °C for 6 h. Then the seeds were redried to their original moisture content, which took 10–12 days. The seeds were kept in the dark for 2 days before use. The main result of their study revealed that seed priming with P-85-SWCNTs in the concentration range of 0.4–100 mg L^–1^ stimulates photosystem II photochemistry.

The seeds chosen by Chen et al. were of maize (*Zea mays* L.) from two varieties, Yuebaitiannuo7 and Yuecainuo2.^70^ The authors studied the effects of MWCNTs on the seed germination and early growth of maize under cadmium stress. Tests were conducted in three different cadmium concentrations: 0, 100, and 200 mg L^–1^, and three MWCNTs: 0, 100, and 200 mg L^–1^. The maize seeds were surface sterilized with a 3% NaClO solution for 20 min, followed by washing with distilled water. The seeds were soaked in the MWCTN solution for 24 h, and the solution was replaced after 12 h. In the study, they observed that inhibition of germination by cadmium in maize seeds might be genotype-specific. Cadmium (100 mg L^–1^) reduced the germination in Yuecainuo2, but no significant effects were observed for Yuebaitiannuo7. Multiwall carbon nanotubes promise to mitigate cadmium toxicity by promoting seed germination and modulating the antioxidant defense system. They have potential applications as seed-soaking reagents and seed coating materials in agricultural production systems to address the challenges of heavy metal-contaminated soils.

Gorczyca and co-workers evaluated the effect of MWCNTs on pea seeds and their influence on the morphology, germination coefficient, infection of seedlings by naturally occurring microorganisms, infection caused by *Fusarium culmorum* (FC), and efficiency of photosystem II.^71^ The pea variety chosen for the tests was Tarchalska. MWCNT suspension was prepared at a concentration of 1 mg mL^–1^. The seeds were placed in Erlenmeyer, and after shaking for 24 h, all seeds in the flasks were rinsed three times with sterile distilled water. The results of the Gorczyca study showed that MWCNTs limit the germination of pea seeds. MWCNTs also reduced the pathogenesis of seedlings caused by FC and organisms of the natural seed microbiome.

In another study involving the utilization of MWCNTs,^72^ results showed that the effect of MWCNTs was not statistically significant compared to the control on the growth of castor seeds (*Ricinus communis L*.). In their work, the authors investigated the effect of ten different concentrations of MWCNTs on the germination and growth of the seeds The concentrations of MWCNTs were 2, 5, 10, 20, 50, 75, 100, 125, 250, and 500 μg mL^–1^. The seeds were placed in Petri dishes, and the MWCNTs were added. Carbon nanotubes (CNTs) had toxic effects at high concentrations but promoted rice seed germination and root growth at lower concentrations.^73^ The concentrations of carbon nanotubes on the tests were 50, 100, and 150 μg mL^–1^. The rice seeds were inoculated on standard agar Murashige and Skoog medium with concentrations of CNTs. The seeds were sterilized with 0.1% mercuric chloride for 5 min, then rinsed five times with sterile water. The first result observed in the work was that germination started on the first day compared to the control for the seeds treated with carbon nanotubes. This result suggests a stimulation of the rice seed germination by the carbon nanotubes.

Wenli et al. reported the effect of single-walled carbon nanotubes (SWCNTs) on seed germination and physiological changes under drought conditions in soybean seeds (*Glycine max L. Merrill*).^74^ The concentration of SWCNTs used on the tests was 1 mg L^–1^. The soybean seeds variety Zhonghuang 35 was surface sterilized with a 5% sodium hypochlorite solution for 5 min, then rinsed with deionized sterilized water. The seeds were soaked for 30 min in the SWCNT solution and then dried on a superclean bench. The seeds were placed in Petri dishes, and different PEG 6000 concentrations were added. The major results indicated that the SWCNTs promoted the germination of soybean seeds under drought stress and decreased the contents of H_2_O_2_ from the seed. On the basis of the studies reported in this subsection, it is possible to see the potential application of carbon nanotubes (single- and multiwalled) as priming agents and their importance for the development of a sustainable agricultural system.

Further research is needed to determine the possible effects of CNTs on the environment and human health because their toxicity is still up for debate. In this context, creating novel applications and addressing potential safety issues require a thorough understanding of the underlying characteristics and behavior of CNTs. This understanding necessitates an interdisciplinary strategy encompassing engineering, materials science, physics, chemistry, and biology.

#### 3.2.2 Graphene

Graphene, a two-dimensional carbon allotrope, has attracted much scientific attention due to its outstanding qualities and possible uses.^75^ Graphene has a crystalline structure with a 2D atomic plane similar to a honeycomb network. Graphene may serve as a fundamental building block capable of constructing various carbonaceous structures, such as carbon nanotubes, for instance.^76^ Graphene is nontoxic to various species, such as plants, bacteria, vertebrates, and fungi.^77,9^ The most current developments in graphene research focus on the distinctive properties of the material, production techniques, and countless potential uses.^78^

Pandey et al. reported the effects of graphene on biomass accumulation in bioenergy crops.^11^ The graphene used in the study had three layers with lateral dimensions of 1–2 μm. Different concentrations of graphene were used: 50, 100, 200, 500, and 1000 μg mL^–1^ supplemented in the Murashige and Skoog medium. The seeds chosen for the tests were sorghum (*Sorghum bicolor* L.) and switchgrass (*Panicum virgatum* L.). The authors found graphene in concentrations of 50 and 200 μg mL^–1^ had effective stimulation for germination of both bioenergy crop species. For switchgrass seeds, concentrations of 50 μg mL^–1^ had a more significant effect than 200 μg mL^–1^. The exposure to graphene at a concentration of 50 μg mL^–1^ resulted in a 15% increase in the germination rate. Similar results were observed for the sorghum seeds.

Zhang and co-workers studied the effect of a lower concentration of graphene on tomato seed and seedling growth.^79^ The seeds chosen for the tests were tomato seeds (*Solanum lycopersicum*) called “Nugget Hybrid”. The seeds were placed in cotton-cushioned glass bottles with 5 mL of graphene solution (40 μg mL^–1^). The notable discovery from applying graphene to tomato seeds was that it significantly hastened the germination process, with a germination rate of 33.3% compared to the 6.7% of the control. Furthermore, the root of graphene-treated seeds was longer than that of control seeds. This increase in the germination rate could be associated with the penetration of graphene in the seed husk, which may cause an increase in water uptake during the early stage of germination. Overall, their study demonstrated the positive effects of graphene on the germination of tomato seeds. The ability of graphene to penetrate the seed husks plays a crucial role in facilitating water uptake, thereby promoting rapid seed germination and increasing the percentage of germination rates.

Cakir and co-workers investigated the effects of graphene on the germination and seedling growth of wheat and barley.^78^ Seed preparation involves soaking the seeds in different graphene concentrations: 0, 500, 1000, and 2000 mg L^–1^ overnight at 25 °C in the dark. The seeds were placed on wet cotton and exposed to the solution with and without graphene. The study revealed that the use of a 2000 mg L^–1^ graphene solution had the most significant effect on seed germination, with the influence varying based on graphene concentration. The presence of graphene led to a decrease in both root and shoot weight. Furthermore, after 5 days, graphene exhibited a detrimental effect on the root and shoot length of barley, ultimately inhibiting its germination process.

The number of works reported in the literature on seed priming with graphene is still low, but it can be seen that graphene can impact different species in a dose-dependent manner.

#### 3.2.3 Graphene Oxide

Graphene oxide (GO) is a 2D material with outstanding properties and the potential to be used in biological applications. However, few studies have been conducted on the impact of graphene oxide in agriculture, especially in the field of seed priming. As an example, GO can increase the activity of antioxidant enzymes, manage hormone pathway genes (ARR3, LAX3, ABCB1, and PIN7), and regulate genes related to root growth and development (AARO1, ARF19, and TTG1) in the “Gala” apple.^80^ Carbonaceous nanomaterials have been reported to affect seed germination and root growth in tomatoes and corn,^81,82^ increase seed germination in rice, and improve the vitality and health of rice seedlings.^83^ These findings indicate that graphene oxide may play an important role in enhancing seed germination and promoting plant growth in different crops. This discovery makes it an intriguing and promising field for further research and exploration.

According to Guo,^84^ GO-treated seeds had significantly stronger shoots compared to the control seeds. As reported, they treated tomato seeds with graphene oxide and investigated the effects of its concentration on shoot and stem growth in terms of morphological and biochemical indices. The researchers used a graphene solution and modified the surface using COOH and C–OH groups to increase dispersibility in an aqueous medium. The seeds used in the tests were from the cultivar *Solanum lycopersicum*. The seeds were treated with graphene oxide solutions at concentrations of 0, 20, and 50 mg L^–1^. The number of cortical cells, as well as the cross-section area and diameter, were significantly increased. The findings indicated that exposure to GO resulted in a dose-dependent increase in the total length and surface area of seedling roots. This fact suggests that GO may potentially enhance nutrient absorption capacity in plants. Additionally, the dry weight of the roots was significantly higher in seedlings treated with graphene than in the control group. These results suggest that the increased root growth and biomass accumulation could be attributed to the effects of GO on the physiology of the plants. This study highlights the potential of GO for improving plant growth and development.

Salinity stress is an important abiotic factor related to environmental aspects that limit plant growth and development. Kaymak et al.,^13^ described the use of graphene oxide as a seed priming agent for seeds of melon (*Cucumis melo* L.) under salinity conditions for the first time. In the experiments, the researchers used three different melon cultivars found in Turkey: Hasanbey, Kirgakağaç-589, and Kirgakağaç-637. To determine the effect of GO on seeds, five doses were applied (0, 0.25, 0.50, 0.75, and 1.0 mg mL^–1^) in the experiments. The NaCl solutions were prepared with sterile distilled water at concentrations of 0, 5, 10, and 15 g L^–1^. For the priming procedure, the seeds were shaken in a 5% sodium hypochlorite solution for 2 min, immersed in GO solutions, and primed in a rotary shaker at 150 rpm in the dark at room temperature for 24 h. After priming, the seeds were dried at room temperature for 24 h. One first conclusion obtained from the study was that the germination percentage of melon seeds decreased with increasing NaCl concentrations. On the basis of the experiments, primed melon seeds with GO showed higher germination percentages than the control group in all treatments. GO priming was more significant in the presence of salinity stress. However, the effective dose of GO varied depending on the cultivars. For example, in a dose-dependent manner, the highest germination percentage of Hasanbey, Kirgakağaç-589, and Kirgakağaç-637 at 10 g L^–1^ NaCl was determined as 0.75, 0.50, and 0.25 mg mL^–1^ GO, respectively.

The research conducted by Yin and co-workers revealed that GO and cadmium alone, and the coexistence of both in the suspension had different influences on the germination of rice seeds. Their work was based on the impact of graphene oxide on the germination of rice (*Oryza sativa* L. cv. Changyou 99–1) in a simulated environment contaminated with cadmium.^85^ Cadmium, being a toxic heavy metal can lead to inhibition and abnormalities in the growth of various plant species. The metal interferes with the physiological and biochemical processes of plants, hindering photosynthesis and respiration and causing permanent damage to cellular structures. Additionally, cadmium poses a significant risk to public health.^86−88^ To study the influence of GO and its effects on cadmium uptake, different concentrations of GO (0, 100, 500, and 1500 mg L^–1^), Cd^2+^ (0, 5, and 20 mg L^–1^), and four sampling times (0.5, 1, 5, and 20 min) were applied. The rice seeds were surface-sterilized with a 2% H_2_O_2_ solution for 15 min and then washed with deionized water. The seeds were placed in a Petri dish with a double-layer filter paper, and the solutions (GO and cadmium) were placed on top of it. The presence of GO suspension significantly decreased the cadmium concentration in the solution, which may indicate that GO has a great adsorption capacity on Cd^2+^. Therefore, GO may change the effects of Cd^2+^ on plant growth.

Furthermore, this study revealed notable interactive impacts of GO and Cd^2+^ on cadmium concentrations in plant roots and shoots. The findings suggested that the presence of GO can modify the influence of Cd^2+^ on seed germination, seedling development, and the absorption of Cd^2+^.

A recent study by Lee and co-workers,^89^ reported on the impact of graphene oxide on the germination and early stages of growth in lettuce, radish, perennial ryegrass, alfalfa, and cucumber seeds. The germination rates and growth of these cultivars were analyzed after the exposure of seeds to GO at concentrations of 0, 0.2, 0.4, 0.8, and 1.6 mg mL^–1^. The study demonstrated that the germination rate of lettuce decreased as the concentration of GO increased, while no significant effects were observed on the germination rate of other plants. In contrast, the presence of GO had a negative impact on the growth of lettuce, alfalfa, and radish. Specifically, when exposed to 1.6 mg mL^–1^ of GO, the shoot and root length of lettuce decreased by 87% and 86%, respectively. These findings suggest that high concentrations of GO may impede the germination and early growth of plants, depending on species and material dose. Thus, their results could potentially contribute to the development of regulatory guidelines for the safe disposal of nanomaterials.

Kim et al.,^90^ studied the effects of silver-graphene oxide on seed germination and early stage growth in alfalfa (*Medicago sativa* L.), radish (*Raphanus sativus* L.), and cucumber (*Cucumis sativus* L.). In the study, the seeds were treated with a suspension of Ag-GO at concentrations of 0.2–1.6 mg mL^–1^. Before the exposure to Ag-GO, the seeds were sterilized with a 10% sodium hypochlorite solution for 10 min and rinsed with distilled water. Then, the seeds were transferred onto filter paper and placed in a Petri dish containing 5 mL of Ag-GO suspension at different concentrations (0, 0.2, 0.4, 0.8, and 1.6 mg mL^–1^). As we well-known, the effects of nanomaterials on crops are dose-dependent. In the study, the impact of Ag-GO was observed on the plant species at an early stage of development. For alfalfa, root elongation was observed; radish showed inhibited root elongation, and cucumber was not affected. In summary, the treatment with Ag-GO inhibited shoot growth in cucumbers and root growth in alfalfa but enhanced both root and shoot growth in radish. The uptake of Ag by seedlings was dependent on the dose of Ag-GO. As the concentration of Ag-GO increased, the production of H_2_O_2_ also increased, which may explain the inhibited growth of alfalfa seedlings at high levels of H_2_O_2_.

Anjum and co-workers conducted a study to investigate the effect of different concentrations of single-bilayer graphene oxide sheets on the germination of fava bean (*Vicia faba L*.).^91^ In brief, the concentrations of GO suspensions used in the tests were 0, 100, 200, 400, 800, and 1600 mg L^–1^. First, the seeds were immersed in a 10% sodium hypochlorite solution for 10 min, then placed in a Petri dish on filter paper and soaked in a 4 mL solution of GO in different concentrations. Seed germination rate and root length were significantly impacted by the GO concentration. A decrease in germination rate and root length was observed for seeds treated with concentrations of 100, 200, and 1600 mg L^–1^ of GO. An increase in those characteristics was observed for seeds treated with GO at concentrations of 400 and 800 mg mL^–1^. According to this study, the impact of GO on faba was dose-dependent; at concentrations of 400 and 800 mg mL^–1^, the effect of GO was positive but negative at concentrations of 100, 200, and 1600 mg mL^–1^ compared to the control. Applying GO as a seed priming agent significantly improved the health of fava beans. This is supported by increased seed relative water content, enhanced activity of H_2_O_2_-decomposing enzymes (APX and CAT), and higher levels of osmolytes. These effects collectively reduced oxidative stress, improved cell membrane integrity, and reduced protein oxidation, resulting in better health. This improvement was reflected in increased seed germination and root elongation.

Sankaranarayanan et al. reported a synthesis of a composite based on graphene oxide and carbon dot (CD) using brown macroalga (*Macrocystis pyrifera sporophyte*) bio-oil as a precursor.^92^ The cultivar chosen for the test was the seeds of mung bean (*Vigna radiata* L.), a Leguminosae widely cultivated in Asia. The selected seeds were soaked in different concentrations (vol %) of water/GO-CD in the following proportions: 100/0, 75/25, 50/50, and 25/75. Germination experiments were conducted in plastic boxes filled with soil from the VHNSN College campus. Results were best obtained with a 75/25 vol % water/GO-CD ratio. Seeds treated with this material proportion resulted in better germination on day 5, but an increase in GO-CD concentration (>25 vol %) resulted in lower germination. The decrease in germination rate may be due to the higher concentrations of CDs filling the active pores in mung bean.

#### 3.2.4 Fullerenes

Fullerenes are distinct closed-cage carbon compounds, with C60 and C70 being the most accessible. They have diverse characteristics and have sparked great attention since their discovery in 1985.^93^ Fullerenes are structurally similar to soccer balls, with carbon atoms organized in pentagons and hexagons. They have electrical conductivity, mechanical strength, and unique thermal conductivity qualities. Fullerenes are chemically reactive and may create derivatives with various functional groups, broadening their applicability.^94^ Furthermore, they exhibit optical characteristics appropriate for optoelectronic devices. Fullerenes show promise in various sectors, including nanotechnology, materials science, electronics, and medicine, as well as new approaches in agriculture.

Due to the possibility of functionalization and chemical modification, some fullerene derivatives exist. Kong and co-workers utilized a soluble fullerene derivative for agricultural applications.^95^ They conducted research about the application of fullerol [C_60_(OH)*_n_*] to wheat seeds. Fullerol was dissolved in a PEG-6000 (20%) solution, and the concentrations of fullerol were 0, 25, 50, 100, 200, and 300 mg L^–1^. The solution of fullerol was added directly to the seeds in a Petri dish. The effects of fullerol on wheat seed germination and seedling growth under drought stress conditions were evaluated. Results showed that fullerol, particularly at concentrations ranging from 25 to 200 mg L^–1^, significantly enhanced seed germination in two wheat cultivars. The most effective concentration was found to be 50 mg L^–1^. Furthermore, seed priming with 50 and 100 mg L^–1^ of fullerol demonstrated pronounced effects on promoting seedling growth and enhancing the activities of antioxidant enzymes (SOD, CAT, and POD) while reducing the accumulation of ROS and MDA in both wheat cultivars under drought stress. Polyhydroxyl fullerenes are known to be sponges for free radicals. Shafiq and collaborators investigated the hypothesis that polyhydroxyl fullerene nanoparticles (PHF) can dilute oxidative stress in crops exposed to salinity.^96^ In another study, fullerol was used on *Brassica napus* under water stress conditions.^97^ The seeds were treated with a PEG-6000 (15%) solution with different concentrations of fullerol (0.01–1000 mg L^–1^). Fullerol in the range between 0.1 and 100 mg L^–1^ had the best results for germination tests. On the other hand, there were no positive effects under water stress for concentrations above this range. Adding fullerol to water-stressed plants improved seed germination, aboveground biomass, photosynthetic metrics, ABA accumulation, and antioxidant levels, reducing oxidative harm.

Another application of the fullerene soluble derivative (polyhydroxy fullerene), also called fullerenol, in managing abiotic stress was the evaluation of the germination condition of wheat under high salinity conditions.^98^ The findings of this research were that fullerenol nanopriming enhanced net primary production in salt-stressed wheat by regulating oxidative stress and tissue ionic balance. Salinity stress altered wheat growth patterns, increasing the Leaf Area Ratio (LAR) and Relative Growth Rate (RGR) while reducing Net Assimilation Rate (NAR). Fullerenol nanopriming mitigated these changes. Additionally, salinity stress decreased shoot and root biomass, ion absorption, and chlorophyll levels in wheat plants, which were improved with fullerenol therapy. Despite increased enzyme activity, salt-stressed wheat plants exhibited elevated levels of reactive oxygen species (ROS), such as O^2–^ anion and H_2_O_2_, alongside heightened lipid peroxidation (MDA levels). However, fullerenol therapy effectively reduced these oxidative stress indicators.

Table 1 summarizes the effects of some nanomaterials on different crops.

**Table 1:** Reported Nanomaterials for Crop Development and Growth

nanomaterial	crop	concen. (mg L^–1^)	seed steril. method	main effect	ref
AgNPs, AuNPs	*Allium cepa* L.	AgNP 31.3, AuNP 5.4		Enhancement of germination, leaf length, plant height	(99)
F_2_O_3_ NPs	*Triticum aestivum* L	25–600	3 min, 10% sodium hypochlorite	Enhancement of germination and shoot length	(100)
Si NPs	Wheat	0–1200	2 min, 2.6% sodium hypochlorite	Stimulation of chlorophyll contents and antioxidant enzyme	(101)
CuO NPs	*Phaseolus vulgaris* L.	0–1000	10 s, 10% sodium hypochlorite	Promotion of seedling weight	(102)
Carbon nanotubes	*Solanum lycopersicum L*.	10–40	10 min, 50% Chlorox solution	Improved water uptake inside of the seeds	(16)
Multiwalled carbon nanotubes	*Ricinus communis* L.	2–500		Stimulation on the biomass and root growth	(72)
Carbon nanotubes	*Oryza sativa* L.	50–150	5 min, 0.1% mercuric chloride	Promotion of germination and root growth	(73)
Carbon nanotubes	*Lycopersicum esculentum* L., *Allium cepa* L., *Brassica rapa* L., *Raphanus sativus* L.	10–40	10 min, 50% sodium hypochloride	Improved germination rate	(66)
Multiwalled carbon nanotubes	Pea	0–1000	10% sodium hypochlorite	Increment of the length of the shoots and roots	(71)
Graphene	*Sorghum bicolor* L., *Panicum virgatum* L.	50–200	2 min, 70% ethanol	Improvement in salt stress response	(11)
Graphene	Wheat, Barley	0–2000		Decrease in root and shoot weight	(78)
Graphene	Rice	0–200	10 min, 3% hydrogen peroxide	Inhibitions effects	(103)
Graphene oxide	*Cucumis melon* L.	0–1000	2 min, 5% sodium hypochlorite	Improvement in salinity stress response	(13)
Graphene oxide	*Vicia faba* L.	0–1600	10 min, sodium hypochlorite	Decreased oxidative stress, improved relative water content	(91)
Graphene oxide	*Zea mays* L., *Oryza sativa* L.	0–1500	15 min, 2% hydrogen peroxide	GO reduced the inhibitive effects of Cd^2+^	(85)
Graphene oxide	*Solanum lycopersicum* L.	0–200		Improved root development and auxin content	(84)
Polyhydroxy fullerene	Wheat	10–120 (nM)	1 min, 0.1% sodium hypochlorite	Salinity tolerance	(96)
Fullerenol	*Triticum aestivum* L.	25–200	15 min, 2% sodium hypochlorite	Increase the germination under drought stress	(95)

In agreement with the reported works, it seems that applying different nanomaterials can boost the development of seeds and seedlings of different cultures, improve seedling vigor, and increase stress tolerance. All these improvements indicate the potential of carbon nanomaterials in seed priming to build a more efficient agricultural process.

## 4 Carbonaceous Materials as a Tool for the Improvement of Crop Yield and Nutrient Uptake

A variety of nanomaterials have been applied in agriculture. Carbonaceous materials can be obtained from different sources, such as the exfoliation of graphite or organic matter. This second option leads to the development of sustainable carbonaceous materials derived from biochar, which have gained recognition for their potential to improve nutrient uptake, soil health, and plant growth.^9^ Carbonaceous materials, like graphene oxide (GO), can be used in many applications with the purpose of increasing crop production. GO has been used as a nanoplatform to slow the release of micronutrients, solving the low efficiency linked with conventional fertilizers.^45^ According to Kabiri, using GO as a carrier platform may emerge as a slow-release carrier for sustained delivery of micronutrients such as Zn and Cu, increasing availability during all stages of plant development.^45^ This result shows that GO can be used as a slow-release carrier platform for micro- and macronutrients and be a new tool for crop nutrition, helping to improve sustainable agriculture. Another potential use of carbonaceous materials in agriculture is as carbon nanoparticles (CNPs). Zhao et al. reported a significant enhancement in growth and nutrient uptake by using CNPs in corn plants (*Zea mays* L.).^47^ The evaluations were performed in sandy soil with low organic matter, and the application of CNPs at a concentration of 200 mg kg^–1^ could increase plant height, shoot, and biomass yield by 110%, 313%, and 281% in spodosol soil, respectively. Additionally, the application of CNPs increased the use efficiency of N, P, and K by 1891%, 609%, and 597% in Spodosol soil, respectively. Their results suggest that applying CNPs can be a strong candidate for improving growth, yield, and nutrient uptake. In a study involving *Arabidopsis thaliana* L. and graphene oxide (GO), the number of flower buds was significantly increased by GO.^8^ Another study involving *Zea mays* used the application of carbon nanotubes (MWCNT) on the germination of maize seedling.^104^ The MWCNT affected maize seedlings germination in a dose-dependent manner. Furthermore, MWCNTs at low concentrations improved water content, nutrient transport, and biomass, which were crucial for seedling development. On the basis of the above works, it seems likely that carbonaceous materials are a potential device for improving growth, yield, and nutrient uptake, leading to more efficient fertilizer use and enhancing current agricultural practices.

The special qualities of carbonaceous materials provide opportunities to increase seed germination rates, nutrition availability, and stress tolerance. The development of sustainable agricultural methods, which promote improved crop yield and global food security, would surely benefit from more research into the mechanisms behind these processes and from the improvement of priming techniques. Table 2 presents the effects of applying carbonaceous materials on various plant growth parameters, including plant height, root development, and biomass accumulation. It compares the performance of treated plants against control groups. The data illustrate how different types and concentrations of carbonaceous materials influence these growth metrics, providing insights into their potential benefits and limitations in promoting plant growth and enhancing resistance to environmental stressors.

**Table 2:** Impact of the Application of Carbonaceous Materials on Plant Height, Root Development, And Biomass

carbonaceous nanomaterial	crop species	concentration range(mg/L)	increase in plant height^a^ (%)	increase in root size^a^ (%)	increase in total biomass	ref
Graphene	*Solanum lycopersicum*	0–40	17	12.5	9.4	(79)
Graphene	*Sorghum bicolor*	50–200	2^b^	10^b^	45.8	(11)
Graphene	*Panicum virgatum*	50–200	50^b^	30^b^	28	(11)
Graphene	*Brassica oleracea (cabbage)*	500–2000	–61	–78		(105)
Graphene	*Lycopersicon esculentum (tomato)*	500–2000	–53	–46		(105)
Graphene	*Amaranthus tricolor*	500–2000	–39	–18		(105)
RGO	*Brassica napus*	1000	–26.8	27.7		(106)
GO	*Brassica napus*	1000	–37.7	38.6		(106)
GO	*Solanum lycopersicum*	50–200	20^b^	29^b^		(84)
GO	*Vigna radiata*		20			(92)
GO	*Cucumis melo*	250–1000	94			(13)
GO	*Medicago sativa*	200–1600		–46		(90)
GO	*Raphanus sativus*	200–1600	52	45		(90)
GO	*Cucumis sativus*	200–1600	–16			(90)
SWCNT	*Lycopersicum esculentum*	0–40	93	83		(66)
SWCNT	*Allium cepa*	0–40	69	90		(66)
SWCNT	*Raphanus sativus*	0–40	54	61		(66)
SWCNT	*Brassica rapa*	0–40	9	–15		(66)
MWCNT	*Triticum aestivum*	0–90	29.5	75.8	34	(107)
SWCNT	*Glycine max*	1	10	15	5	(74)
MWCNT	*Zea mays*	0–200	31.1	8.37		(70)
MWCNT	*Pisum sativum*	250	24	15		(71)
MWCNT	*Oryza sativa*	0–150	13	9		(73)

a The increase was expressed using values of mass or size variation.

b Estimated values.

## 5 Toxicity, Soil Accumulation, and Microbiota Impacts of Carbon-Based Materials

The final subsections highlighted the effects of applying carbon-based materials such as graphene, graphene oxide, fullerene, and carbon nanotubes as seed priming agents. This section will further discuss the potential consequences of these materials on soil health, including their toxicity to soil organisms, seedlings, and microbial communities. The size of the nanomaterial plays a crucial role in its mechanism of action. Nanomaterials smaller than 20 nm can freely penetrate cells, while larger nanoparticles primarily interact with cell wall and membrane proteins. According to studies by Cherati and McGehee,^108,109^ the application of carbon nanomaterials can restore a wide range of genes that are damaged by abiotic and biotic stressors by altering metabolic pathways and inducing the expression of stress-related signaling genes. Interaction between carbon-based materials and proteins from cell wall can induce the generation of reactive oxygen species (ROS),^110^ triggering the activation of hydrolase enzymes (PLC) and calcium-dependent proteins (CDPK), which are involved in antioxidant pathways.^19,110^ Additionally, these interactions can activate hormone signaling pathways, leading to the production of salicylic acid, jasmonic acid, and abscisic acid. These major mechanisms of interaction between carbon-based materials and plant components can be exploited as tools to prevent and mitigate biotic and abiotic stress conditions.

One of the key factors determining the toxicity of carbon-based materials is their concentration, which varies depending on the type of application and crop species. An appropriate concentration of carbon materials can induce the production of specific metabolites, thereby enhancing plant metabolic pathways. Seedling development is a critical stage that can be either benefited or adversely affected. Gao and colleagues^111^ reported that the presence of graphene oxide (GO) exacerbated cadmium (Cd) phytotoxicity in wheat seedlings. Their study demonstrated that seedlings treated with GO and Cd simultaneously were more adversely affected compared to treatments with each compound independently. The study also showed a dose-dependent effect, with GO concentrations ranging from 5 to 40 mg/L, in the presence of Cd, inhibiting total root length, total root surface area, and the number of root hairs in wheat seedlings. Begum et al.^105^ investigated the effects of graphene on cabbage, tomato, lettuce, and spinach seedlings, finding that root and shoot development, cell shape, cell death, and ROS accumulation were directly affected by graphene application. The seedlings exposed to graphene showed significant inhibition of plant growth and biomass accumulation, attributed to ROS overproduction induced by graphene. In contrast to these findings, Lopéz-Vargas et al.^10^ demonstrated that the application of carbon nanomaterials, such as carbon nanotubes and graphene, increased the content of phenols, vitamin C, and H_2_O_2_, as well as the activity of enzymes such as superoxide dismutase, catalase, ascorbate peroxidase, and glutathione peroxidase. The application of these materials resulted in an increase in plant height by more than 40%, as well as increases in stem diameter, fresh shoot biomass, and fresh root biomass compared to control plants that did not receive nanomaterials. Villagarcia et al.^112^ reported a correlation between the level of agglomeration and surface chemical groups of carbon nanotubes and the physiological response of tomato seedlings, with plants showing positive growth responses to well-dispersed and negatively charged multiwalled carbon nanotubes (MWCNTs). These studies highlight that the application of carbon nanomaterials can have either beneficial or detrimental effects on crops, depending on concentration and specific conditions. However, the full mechanisms and final assessment of carbon-based material toxicity in seedlings require further investigation.

Another aspect to consider is the soil accumulation of carbon-based materials and their impact on microbial communities. Biochar, a widely applied carbon-based material, has been used as a soil amendment and for soil remediation.^113,114^ Zhao et al.^115^ explored the effects of GO application to soil and its impact on *Paeonia ostii* under drought stress. They found that GO had no toxicity to *Paeonia ostii* and alleviated drought stress by decreasing ROS accumulation, increasing antioxidant enzyme activities, and enhancing soil water retention. Furthermore, the study noted that GO did not accumulate in the crop due to electrostatic repulsion between the roots and GO.

The combination of carbon nanomaterials with fertilizers can also significantly impact soil biochemistry. Studies have shown that the application of low concentrations of carbon nanomaterials in conjunction with fertilizers improved soil fertility and biochemical properties, thereby boosting the growth and yield of lettuce.^116^ In a study by Oyelami et al.,^117^ the addition of carbon nanomaterials such as fullerene (C_60_), single-walled carbon nanotubes (SWCNTs), and multiwalled carbon nanotubes (MWCNTs) showed varying effects on microbial activity in soil. While overall microbial activity was not significantly affected in the short term, high concentrations of MWCNTs were found to reduce microbial biomass, indicating potential stress effects on soil microorganisms and possible disruptions in nutrient cycling. The study emphasized that the observed effects were based on a short-term experiment (21 days), and long-term impacts of carbon nanomaterials on soil microbial activity remain uncertain. Further research is needed to understand the long-term consequences of CNM exposure in various soil types and conditions. In a study conducted by Wu et al.,^117^ the short- and long-term impacts of C_60_, SWCNTs, and graphene on soil bacterial communities were evaluated. Long-term exposure (360 days) to CNMs, particularly SWCNTs and graphene, significantly decreased the alpha diversity of soil bacterial communities compared to short-term exposure (30 days), suggesting that prolonged exposure reduces bacterial species diversity. Long-term exposure also more significantly altered the beta diversity of soil bacterial communities, indicating that the composition of bacterial communities becomes more distinct and less similar over time with prolonged CNM exposure. The study highlights the need for further research to understand these long-term impacts and to predict microbial responses and potential consequences for terrestrial ecosystem services. Overall, while short-term exposure to carbon-based materials may enhance the diversity of soil bacterial communities, long-term exposure tends to decrease diversity and alter community composition, potentially leading to negative consequences for soil health and ecosystem stability.

## 6 Conclusions

On the basis of the presented works, it can be seen that nanomaterials, like carbonaceous ones, are a promising way to improve the current agricultural system, leaving behind the old practices from the Agrotech revolution. Additionally, seed priming is one of the approaches that can be explored to build a more efficient and sustainable agriculture. Applying carbonaceous materials like graphene, graphene oxide, and carbon nanotubes as seed priming agents can increase the efficiency of agrochemicals against biotic and abiotic stresses. The combined impact of these factors can culminate in less resource use and escalating food production. These findings highlight the potential avenues for future research in applying carbonaceous materials for seed priming. The observed increase in crop growth and development suggests a positive impact on sustainable agriculture. However, it is imperative to address the critical role of nanomaterial concentration, which varies among different species of the same crop. To advance our understanding, future studies should expand their focus to include diverse species, systematically examining the influence of nanomaterial concentrations on each. Moreover, the current predominance of bench-scale studies underscores the need for research to transition beyond laboratory settings. A comprehensive evaluation of the real impact of carbonaceous materials as seed priming agents should encompass the entire growth cycle, from germination to harvest. This targeted approach will contribute significantly to unraveling the nuanced effects and potential applications of carbonaceous materials in promoting sustainable agricultural practices.
